# Research contributions by Indus Hospital Health & Network faculty

**DOI:** 10.12669/pjms.40.2(ICON).9129

**Published:** 2024-01

**Authors:** Shaukat Ali Jawid

It is heartening to be a part of the Indus Hospital Health & Network and publish a special supplement to show case the research being conducted by healthcare professionals affiliated with this institution. This is the third supplement that we have published on the occasion of its forthcoming 7^th^ Biennial Conference scheduled to be held from January 20^th^ to January 28^th^ 2024. The Theme of the conference is “Game Changing Solutions in Healthcare”. This issue contains fourteen original articles, one short communication, six case reports and two manuscripts in the correspondence section.

After a meeting with the scientific committee and those looking after this academic project, some guidelines for the manuscript submission were shared with them. It was decided that they will provide the finalized manuscripts which must be reviewed by at least two reviewers. The manuscripts will be submitted to us along with two peer review reports of each manuscript, Ethics Committee/IRB approval and letter of undertaking signed by all the listed authors. We at our end will also review the manuscripts which will also be checked for plagiarism and similarity score before they are accepted for publication. However, the overall responsibility for the contents of the supplement will be of the Guest Editor representing Indus Hospital. Since we already had published two supplements for Indus Hospital in the past and in view of the past experience we were expecting a smooth sailing this time but it did not happen. However, after having another meeting, we managed to move forward in this project. Still over a dozen manuscripts had to be further revised before they were finally accepted for publication and this whole process took over six months.

We are sure all this must have proved an excellent opportunity to all those involved with this project as well the authors to enhance their professional capacity and further improve their writings. They must have also learnt the whole process through which a manuscript goes before publication. There is no short cut and it takes some time for peer review and final publication after submission.

Indus Hospital Health & Network and its faculty deserve to be commended for their contributions which will definitely further promote the research culture in all its affiliated healthcare institutions in the country. Our hospitals are a goldmine of data but unfortunately most often it is neither documented nor published. The main reason for this is that most of this data is not stored properly and computerized. Indus Hospital did well to install Electronic Medical Records (EMR) system which has made it possible for the researchers to have easy access to all this data. While the world is now moving for more comprehensive Electronic Health Records (EHR) systems, we have failed to even install EMR at our public hospitals which should be one of the priority of the government if they wish to ensure that Pakistan’s contribution to world medical literature is increased. Healthcare professionals need proper resources and conducive environment to undertake research.

Manuscripts included in this supplement cover a wide range of interesting topics. Dr. Masood Alam and his colleagues have highlighted the fact that initial waves of Covid19 Pandemic resulted in higher Intensive Care Unit admissions and also had a higher mortality. This suggested the need for improving early response and resource allocaitons^1^. Faridah Amin and colleagues did a cohort analysis of diabetes and its associated factors. The conclusions of their study highlighted the known risk factors like obesity, hypertension, raised creatinine, microalbuminuria, high LDL and Triglycerides. This will be extremely helpful for the healthcare professionals to take evidence based decisions while planning about prevention and managing diabetes.^2^ Aisha Syed Wali and colleagues in their study showed that symptomatic pregnant women with Covid19 infection had an increased risk of adverse feto-maternal outcome. 3 Epidemiological studies are extremely important for planning, this aspect has been highlighted by Wasfa Farooq and colleagues which shows the clinical characteristics of adult patients presenting at a low resource tertiary care emergency department in the country.^4^

Nida Ghouri has highlighted the potentially valuable tool Full Cup Test(FCT) for assessing the pain severity in a diverse range of patients.^5^ Yet another important study by Madiha Siddiqui and colleagues included in this issue highlights the importance of trained nurses who can play a useful role in providing holistic and timely care for patients suffering from Asthma and Chronic Obstructive Pulmonary Disease(COPD).^6^

We are extremely grateful to the management of Indus Hospital Health & Network for providing us this opportunity to be a part of International conference and highlighting the research work being performed by the faculty at various healthcare facilities affiliated with their institution.

## References

[1] Alam M, Razzak A, Gilani MHS, Shad ZS, Hassan S (2024). Comparison of clinical characteristics, hospital treatment and outcomes in allfour waves of Covid-19 patients at RTEH Muzaffargarh, Pakistan. Pak J Med Sci.

[2] Amin F, Imran M, Hafeez SA, Zehra B (2024). Diabetes and its associated factors:A Retrospective cohort analysis of a large database at Indus Hospital Health &Network. Pak J Med Sci.

[3] Wali AS, Ali MM, Bibi R, Rahim A (2024). The clinical manifestations and pregnancy outcomes of COVID-19 infection at a tertiary care hospital. Pak J Med Sci.

[4] Farooq W, Kazi K, Saleem SG, Ali S (2024). Epidemiology and clinical characteristics of adult patients presenting to a low resource, tertiary care emergency department in Pakistan:Challenges &Outcomes. Pak J Med Sci.

[5] Ghouri N, Mushtaq M (2024). Breaking barriers:Exploring the Full Cup Test (FCT) pain scale at a tertiary care hospital. Pak J Med Sci.

[6] Siddiqui M, Khan F, Saeed S (2024). Task-shifting in asthma and chronic obstructive pulmonary disease management:A review of the obstructive lung disease program. Pak J Med Sci.

